# Changes to physical function and body composition during the first 2 years of polymyalgia rheumatica

**DOI:** 10.1093/rheumatology/keaf375

**Published:** 2025-07-11

**Authors:** Jessica L Leung, Belinda De Ross, Jenny Gianoudis, Natalie Deeble, Victor Yang, David F L Liew, Robin M Daly, Russell R C Buchanan, Claire E Owen

**Affiliations:** Department of Rheumatology, Austin Health, Melbourne, Victoria, Australia; Department of Medicine, The University of Melbourne, Melbourne, Victoria, Australia; Institute for Physical Activity and Nutrition (IPAN), School of Exercise and Nutrition Sciences, Deakin University, Melbourne, Victoria, Australia; Institute for Physical Activity and Nutrition (IPAN), School of Exercise and Nutrition Sciences, Deakin University, Melbourne, Victoria, Australia; Department of Rheumatology, Austin Health, Melbourne, Victoria, Australia; Department of Rheumatology, Austin Health, Melbourne, Victoria, Australia; Department of Rheumatology, Austin Health, Melbourne, Victoria, Australia; Department of Medicine, The University of Melbourne, Melbourne, Victoria, Australia; Institute for Physical Activity and Nutrition (IPAN), School of Exercise and Nutrition Sciences, Deakin University, Melbourne, Victoria, Australia; Department of Rheumatology, Austin Health, Melbourne, Victoria, Australia; Department of Medicine, The University of Melbourne, Melbourne, Victoria, Australia; Department of Rheumatology, Austin Health, Melbourne, Victoria, Australia; Department of Medicine, The University of Melbourne, Melbourne, Victoria, Australia

**Keywords:** polymyalgia rheumatica, physical function, gait speed, frailty, muscle, fat

## Abstract

**Objectives:**

To investigate physical function, body composition and frailty in recently diagnosed polymyalgia rheumatica (PMR) compared with controls.

**Methods:**

In a prospective cohort study, patients with PMR and age- and sex-matched controls were assessed 3 months after glucocorticoid initiation and 18 months later. Measures included the HAQ-DI, grip strength, gait speed, five times chair stand test, Short Physical Performance Battery (SPPB), maximum limb strength/power, habitual physical activity (by accelerometer) and body composition (by DEXA). Frailty was defined as per Fried’s phenotype.

**Results:**

Thirty-six participants with PMR and 32 controls were recruited. Participants with PMR had higher HAQ-DI scores (greater disability) than the controls at both visits (mean difference 0.33 [*P* < 0.001] and 0.39 [*P* < 0.001]). At follow-up, female participants with PMR performed more poorly in the chair stand test (mean difference 3.27 [95% CI: 0.69, 5.84], *P* = 0.02), SPPB (mean difference −1.23 [95% CI: −2.01, −0.45], *P* = 0.003) and gait speed (mean difference −0.24 [95% CI: −0.36, −0.12], *P* < 0.001) than the controls. Between timepoints, female participants with PMR had greater decline in gait speed than the controls (mean difference −0.13 [95% CI: −0.23, −0.03], *P* = 0.009). No significant differences for change in body composition were found. Pre-frailty rates were higher in participants with PMR than the controls (71.2% vs 34.4% (odds ratio 4.7 [*P* = 0.003]) and 60.7% vs 34.4% (odds ratio 2.9 [*P* = 0.04]) at the initial and follow-up visits, respectively).

**Conclusion:**

PMR has a lasting impact on physical function, particularly in females. These findings highlight the need for routine physical function assessments and targeted muscle conditioning measures in PMR management.

Rheumatology key messages

- Patients with PMR have greater functional disability than age- and sex-matched individuals.
- Females with PMR experience a disproportionate decline in gait speed within 2 years after diagnosis.
- Pre-frailty rates are significantly higher in patients with PMR compared to age- and sex-matched individuals.

## Introduction

Polymyalgia rheumatica (PMR) occurs in older adults, giving rise to systemic inflammation with pain and stiffness focused at the shoulder and hip girdle [1]. Even after initiation of standard glucocorticoid therapy, both quantitative and qualitative studies report frequent persistence of symptoms and an incomplete return to premorbid physical health [2, 3].

Retaining independence with activities of daily living (ADLs) is a key priority for patients with PMR, and physical function has been endorsed by an international collaboration of clinicians, researchers and patient partners (Outcome Measures in Rheumatology [OMERACT]) as a core domain that should be routinely measured in PMR clinical trials [4, 5]. Despite its importance, a recent systematic review found that only 25% of studies measured physical function in PMR, most commonly via patient-reported outcome measures [6]. To date, little research has been undertaken concerning the impact of PMR on objective measures of physical function such as muscle strength and physical performance.

Individuals with PMR are conceivably at increased risk for impaired physical function given their older age, inactivity during periods of active disease and long-term glucocorticoid exposure. In addition, pathologic systemic inflammation from PMR likely contributes to muscle-related declines given that chronic low-grade systemic inflammation has been associated with muscle loss, functional decline and is thought to contribute to frailty [7, 8]. Indeed, compared with the expected 9.9% frailty prevalence in a community-dwelling older population [9], disproportionately high rates of frailty (17%) were reported in a cross-sectional study of 41 patients with PMR, with the most common frailty criterion being muscle weakness [10].

Despite the evident risk factors for deteriorating muscle function in patients with PMR, no study, to our knowledge, has comprehensively and longitudinally assessed objective physical function outcomes in a PMR population. Therefore, the aim of this study was to investigate changes to muscle strength, physical function and body composition in patients with a recent diagnosis of PMR, and to compare them to an age- and sex-matched comparator group. Furthermore, frailty and sarcopenia rates were determined in participants with PMR and compared with controls.

## Methods

### Patient population

The study population has been described elsewhere [11]. In brief, patients with a new diagnosis of PMR confirmed by a rheumatologist and meeting the 2012 EULAR/ACR Classification Criteria were recruited from a tertiary hospital in Australia [12]. Age- and sex-matched controls were recruited on a volunteer basis from the community or our existing research database. Exclusion criteria are outlined in Supplementary Data S1.

### Data collection

An initial assessment was undertaken 3 months after PMR cases commenced prednisolone and repeated 18 months later.

#### PMR disease measures

PMR disease activity was evaluated using the PMR-Activity Score (PMR-AS), where a score <7 defines low disease activity [13]. Current prednisolone dose was recorded at each visit, and cumulative prednisolone exposure was calculated from the electronic medical record. Serology (RF, anti-citrullinated peptide antibody [ACPA]) was tested at the initial visit, and inflammatory markers (ESR, CRP) were performed at both visits.

#### Anthropometric and body composition assessment

Height (cm) and weight (kg) were measured using a wall-mounted stadiometer and calibrated electronic digital scales, respectively. A total body dual-energy X-ray absorptiometry (DXA, Lunar iDXA, GE Medical Systems; Encore version 16) scan was used to quantify total body and appendicular (sum of arms and legs) lean mass (ALM, kg), total body fat mass (kg) and percentage body fat. A skeletal muscle mass index (SMI) was derived from ALM divided by height (m^2^).

#### Physical function testing

Functional independence was assessed using the HAQ-DI [14].

Gait speed (m/s) was quantified using the 4-m walk test assessed via timing gates at usual walking pace, with the fastest result from two attempts used. The time (s) to complete the five times chair stand test was used to measure functional lower limb strength. These two tests were combined with an assessment of balance to form the Short Physical Performance Battery (SPPB), a validated composite assessment of lower extremity function with a score ranging from 0 (worst) to 12 (best performance) [15].

Grip strength (kg) was measured using a hand-held device (Jamar dynamometer, Asimov Engineering Co., Los Angeles, CA, United States) (see Supplementary Data S1). Lower limb and upper limb muscle strength were measured using a Keiser pneumatic leg press and seated row machine with A420 electronics fitted, from which the one-repetition maximum (1-RM) weight was recorded (kg). Maximum lower limb power (W) at 40% and 70% 1-RM were also measured (see Supplementary Data S1).

#### Habitual physical activity and sedentary time

Self-reported daily physical activity was assessed using the Physical Activity Scale for the Elderly (PASE) survey [16]. Objectively measured physical activity (average daily step count) and sedentary time (min) were assessed using an accelerometer (ActivPAL3, PAL technologies Ltd, Glasgow, United Kingdom) (see Supplementary Data S1).

#### Frailty

Frailty was defined as per Fried’s phenotype, which is widely considered the gold standard classification approach [17]. Criteria were modified slightly to account for the data available, as is common practice (Supplementary Table S1). Participants who met at least three criteria were classified as frail, whilst those who met one to two criteria were classified as pre-frail.

#### Sarcopenia

Sarcopenia was defined as per the revised European Working Group on Sarcopenia in Older People (EWGSOP2) criteria (see Supplementary Data S1) [18].

### Missing data

Multiple imputation using chained equations was used to handle missing data in outcome variables, with the predictive mean matching method used to impute all variables (see Supplementary Data S1).

### Statistical analysis

All analysis was performed using Stata 17.0 (StataCorp, Texas, United States, 2021). The distribution of datapoints was examined using quantile–quantile plots to assess normality. Participant population characteristics are derived from the raw (non-imputed) data and reported as mean (S.D.) or percentages as appropriate. Habitual physical activity, sedentary time and functional independence (HAQ-DI) were compared between the groups using linear regression analysis adjusting for age and sex. The analyses for physical function and body composition parameters were divided by sex, since inherent differences between sexes were expected. Parameters were compared between the groups at both the initial and the follow-up visit using linear regression analysis adjusting for age. Change scores (the change in parameters between timepoints, from the initial to the follow-up visit) were analysed within each group using linear regression analysis. The differences in change scores between the groups were compared using a linear mixed-effects model. Participant group and time were included as categorical fixed effects and participant ID as a random effect. For participants with PMR, associations between change scores and cumulative glucocorticoid exposure (at the follow-up visit) were examined using linear regression analysis adjusted for age. The proportions of robust, pre-frail, frail and sarcopenic participants in each group were compared using logistic regression analysis adjusted for age and sex. Significance levels were set at *P* < 0.05.

### Ethics approval

The study received ethics approval from the Austin Health Human Research Ethics Committee (reference number HREC/44292/Austin-2018), and all participants gave written informed consent prior to participation.

## Results

### Study population

Thirty-six participants with PMR and 32 controls were recruited from April 2019 to January 2022. The mean age of participants with PMR and controls was 68.7 (S.D. 8.3) and 70.7 (7.8) years, respectively, with 18 females in each group (50% and 56%). There were no major demographic differences between the groups (Table 1). The comorbidity burden was similar [mean (S.D.) Charlson comorbidity index: PMR 3.6 (1.1) and controls 3.1 (1.2)], noting that the presence of PMR alone accounted for 1 point in the index for all participants with PMR. RF was positive in one (2.8%) PMR participant, with a low titre of 20 IU/ml. ACPA was negative in all participants with PMR.

**Table 1.** Participant population characteristics^a^

	Participants with PMR (*n* = 36)		Control participants (*n* = 32)	
	Females (*n* = 18)	Males (*n* = 18)	Females (*n* = 18)	Males (*n* = 14)
Age (years), mean (S.D.)	70.8 (6.7)	66.6 (9.2)	71.0 (7.9)	70.4 (7.9)
Caucasian, *n* (%)	13 (72)	16 (89)	14 (78)	11 (79)
BMI (kg/m^2^), mean (S.D.)	28.6 (5.5)	27.6 (5.5)	27.3 (4.1)	29.3 (3.8)
Underweight, *n* (%)	1 (6)	1 (6)	0 (0)	0 (0)
Normal, *n* (%)	3 (17)	4 (22)	5 (28)	1 (7)
Overweight, *n* (%)	6 (33)	8 (44)	9 (50)	6 (43)
Obese, *n* (%)	8 (44)	5 (28)	4 (22)	7 (50)
Charlson comorbidity index, mean (S.D.)	3.8 (1.0)	3.4 (1.2)	2.9 (1.2)	3.3 (1.1)
Physical activity, mean (S.D.)				
Initial	137 (59)	188 (70)	166 (58)	222 (83)
Follow-up	126 (59)	188 (85)	163 (67)	162 (66)
Average daily step count, mean (S.D.)				
Initial	9361 (3765)	8888 (3722)	9308 (3476)	7350 (3257)
Follow-up	8019 (3976)	7505 (4009)	9394 (2864)	7003 (3072)
Average daily sedentary time (min), mean (S.D.)				
Initial	527.9 (109.5)	565.0 (111.8)	546.1 (148.9)	641.8 (61.6)
Follow-up	544.4 (80.5)	589.4 (95.4)	514.9 (123.8)	673.6 (97.3)

a Based on raw (non-imputed) data.

Two participants with PMR were lost to follow-up (*n* = 1 male; *n* = 1 female): the first due to health issues unrelated to PMR, and the second due to a loss of contact. All other participants completed the follow-up assessment. However, due to strict restrictions in Melbourne (Australia) during the COVID-19 pandemic, not all participants were able to undergo assessments of upper limb (chest press) and lower limb (leg press) muscle strength at both timepoints. Twenty-seven (75%) and 20 (56%) participants with PMR completed these tests at the initial and follow-up visits, respectively, compared with 28 (88%) and 27 (84%) control participants. As previously outlined, multiple imputation analysis was used to account for missing data.

### PMR treatment and disease activity

The median daily prednisolone dose was 10.0 mg (IQR 9.0, 12.5 mg) and 1.5 mg (IQR 0.5, 3.0) at the initial and follow-up visits, respectively. At follow-up, eight (23.5%) patients were also prescribed a steroid-sparing medication (seven prescribed methotrexate and one prescribed leflunomide). At both visits, PMR disease activity was low (median PMR-AS 4.1 [IQR 1.6, 10.2] and 6.4 [IQR 4.1, 14.7] at the initial and follow-up visits, respectively).

### Habitual physical activity and sedentary time

There was no significant difference in the average daily step count nor sedentary time between the groups at either visit (Supplementary Data S2).

### Functional independence

Despite low disease activity, participants with PMR had a significantly higher HAQ-DI than controls at both timepoints, indicating greater functional disability (mean difference 0.33 [95% CI: 0.18, 0.49], *P* < 0.001 at the initial visit and 0.41 [95% CI: 0.24, 0.58], *P* < 0.001 at the follow-up visit). These results remained significant even when the analysis was divided by sex (Tables 2 and 3). HAQ-DI scores did not change significantly in either group over time (Tables 2 and 3).

**Table 2.** Physical function assessments in participants with PMR vs controls (females only)^a^^,b^

	Initial visit^c^	Follow-up visit^c^	Within-group change^d^	Net difference for change^e^
HAQ-DI				
Control	0.08 ± 0.05	0.12 ± 0.04	0.04 (−0.02, 0.10)	0.15 (−0.14, 0.45)
PMR	0.50 ± 0.12	0.70 ± 0.13	0.20 (−0.13, 0.53)	
Adjusted difference^f^	**0.42 (0.15, 0.69)^†^**	**0.57 (0.29, 0.85)^‡^**		
Gait speed (m/s)				
Control	1.32 ± 0.05	1.33 ± 0.04	0.02 (−0.05, 0.09)	**−0.13 (−0.23, −0.03)^†^**
PMR	1.19 ± 0.07	1.12 ± 0.06	**−0.12 (−0.20, −0.04)^†^**	
Adjusted difference^f^	−0.12 (−0.28, 0.03)	**−0.24 (−0.36, −0.12)^‡^**		
Chair stand test (s)				
Control	11.16 ± 0.44	10.68 ± 0.51	−0.48 (−1.12, 0.16)	1.48 (−0.86, 3.83)
PMR	12.90 ± 0.71	13.91 ± 1.20	1.00 (1.56, 3.56)	
Adjusted Difference^f^	1.78 (0.30, 3.26)^*^	3.27 (0.69, 5.84)^*^		
SPPB				
Control	10.5 ± 0.2	10.6 ± 0.2	0.0 (−0.3, 0.3)	−0.2 (−1.2, 0.7)
PMR	9.6 ± 0.5	9.3 ± 0.4	−0.2 (−1.3, 0.9)	
Adjusted difference^f^	−1.0 (−1.9, −0.1)^*^	**−1.2 (−2.0, −0.5)^†^**		
Grip strength (kg)				
Control	22.2 ± 1.3	22.5 ± 1.0	0.4 (−1.2, 1.9)	−1.0 (−4.3, 2.4)
PMR	22.0 ± 1.4	21.4 ± 1.6	−0.6 (−4.0, 2.8)	
Adjusted difference^f^	−0.2 (−4.2, 3.8)	−1.2 (−5.1, 2.7)		
Upper limb 1-RM strength (kg)				
Control	25.4 ± 1.8	26.6 ± 1.3	1.2 (−1.2, 3.6)	1.8 (−2.0, 5.6)
PMR	26.1 ± 1.7	29.1 ± 0.9	3.0 (−0.5, 6.5)	
Adjusted difference^f^	0.6 (−4.4, 5.6)	2.5 (−0.6, 5.6)		
Lower limb 1-RM strength (kg)				
Control	153.1 ± 8.4	155.5 ± 10.2	2.3 (−11.0, 15.7)	9.6 (−19.9, 39.0)
PMR	131.2 ± 16.6	143.1 ± 11.2	11.9 (−23.1, 46.9)	
Adjusted difference^f^	−22.0 (−60.2, 16.1)	−12.5 (−46.1, 21.0)		
Lower limb peak muscle power at 40% 1-RM (W)				
Control	545.4 ± 33.6	571.7 ± 40.8	26.3 (−16.1, 68.7)	96.5 (4.1, 188.9)^*^
PMR	518.3 ± 60.0	641.1 ± 57.7	122.8 (29.4, 216.3)^*^	
Adjusted difference^f^	−28.0 (−172.8, 116.8)	68.1 (−78.2, 214.3)		
Lower limb peak muscle power at 70% 1-RM (W)				
Control	589.7 ± 33.8	578.0 ± 28.2	−11.7 (−74.2, 50.7)	107.7 (−7.4, 222.7)
PMR	541.4 ± 58.4	637.3 ± 62.0	95.9 (−25.5, 217.3)	
Adjusted difference^f^	−49.3 (−191.9, 93.3)	57.9 (−86.5, 202.2)		

a Statistically significant results are highlighted in bold (*P ≤ 0.05, ^†^*P* ≤ 0.01, ^‡^*P* ≤ 0.001).

b HAQ-DI: Health Assessment Questionnaire-Disability Index; SPPB: Short Physical Performance Battery; 1-RM: 1-repetition maximum weight.

c Inferred mean ± standard error in each group from multiply imputed dataset.

d Mean change scores (95% CI) in each group from a linear regression analysis.

e Net difference in change scores (95% CI) from a linear mixed-effects model.

f Mean difference (95% CI) from linear regression analysis adjusted for age.

**Table 3.** Physical function assessments in participants with PMR vs controls (males only)^a^^,^^b^

	Initial visit^c^	Follow-up visit^c^	Within-group change^d^	Net difference for change^e^
HAQ-DI				
Control	0.04 ± 0.02	0.01 ± 0.01	−0.03 (−0.07, 0.02)	−0.04 (−0.20, 0.12)
PMR	0.24 ± 0.06	0.18 ± 0.08	−0.07 (−0.22, 0.09)	
Adjusted difference^f^	**0.23 (0.08, 0.38)^†^**	0.21 (0.05, 0.38)^*^		
Gait speed (m/s)				
Control	1.38 ± 0.05	1.34 ± 0.06	−0.04 (−0.15, 0.07)	0.04 (−0.09, 0.16)
PMR	1.35 ± 0.06	1.33 ± 0.07	0.00 (−0.11, 0.10)	
Adjusted difference^f^	−0.04 (−0.20, 0.12)	−0.01 (−0.21, 0.18)		
Chair stand test (s)				
Control	11.17 ± 0.70	11.57 ± 0.92	0.40 (−0.77, 1.57)	0.63 (−1.37, 2.62)
PMR	11.08 ± 0.63	12.11 ± 1.13	1.03 (−0.79, 2.84)	
Adjusted difference^f^	0.16 (−1.80, 2.12)	1.17 (−1.87, 4.21)		
SPPB				
Control	10.1 ± 0.2	10.2 ± 0.3	0.1 (−0.3, 0.4)	−0.2 (−0.9, 0.4)
PMR	10.3 ± 0.2	10.1 ± 0.3	−0.2 (−0.8, 0.4)	
Adjusted difference^f^	0.1 (−0.5, 0.8)	−0.3 (−1.1, 0.5)		
Grip strength (kg)				
Control	35.8 ± 2.9	33.9 ± 2.9	−1.8 (−4.0, 0.4)	2.5 (−0.1, 5.2)
PMR	34.1 ± 1.9	34.7 ± 2.1	0.7 (−1.4, 2.8)	
Adjusted difference^f^	−4.3 (−9.7, 1.1)	−2.1 (−7.5, 3.4)		
Upper limb 1-RM strength (kg)				
Control	53.5 ± 3.4	51.1 ± 1.7	−2.4 (−7.0, 2.1)	**8.8 (3.8, 13.7)^‡^**
PMR	47.1 ± 2.8	53.4 ± 3.1	**6.3 (2.3, 10.3)** ^†^	
Adjusted difference^f^	**−10.1 (−16.9, −3.3)^†^**	−0.4 (−7.0, 6.1)		
Lower limb 1-RM strength (kg)				
Control	268.5 ± 22.4	260.5 ± 23.0	−7.9 (−27.1, 11.2)	21.2 (−18.4, 60.9)
PMR	208.9 ± 21.9	222.2 ± 19.3	13.3 (−25.0, 51.6)	
Adjusted difference^f^	−72.3 (−137.6, −7.0)^*^	−56.6 (−113.8, 0.6)		
Lower limb peak muscle power at 40% 1-RM (W)				
Control	1065.4 ± 115.1	958.7 ± 102.9	−106.7 (−219.5, 6.1)	**177.4 (62.8, 292.1)^†^**
PMR	895.5 ± 94.7	966.2 ± 105.8	70.7 (−24.4, 165.9)	
Adjusted difference^f^	−272.4 (−528.6, −16.3)^*^	−101.0 (−356.8, 154.8)		
Lower limb peak muscle power at 70% 1-RM (W)				
Control	1224.6 ± 98.2	1077.9 ± 113.4	−146.7 (−358.3, 65.0)	250.8 (32.4, 469.1)^*^
PMR	953.9 ± 95.3	1058 ± 114.2	104.1 (−82.1, 290.3)	
Adjusted difference^f^	**−363.4 (−605.6, −121.2)^†^**	−135.1 (−421.9, 151.6)		

a Statistically significant results are highlighted in bold (*P ≤ 0.05, ^†^*P* ≤ 0.01, ^‡^*P* ≤ 0.001).

b HAQ-DI: Health Assessment Questionnaire-Disability Index; SPPB: Short Physical Performance Battery; 1-RM: 1-repetition maximum weight.

c Inferred mean ± standard error in each group from multiply imputed dataset.

d Mean change scores (95% CI) in each group from a linear regression analysis.

e Net difference in change scores (95% CI) from a linear mixed-effects model.

f Mean difference (95% CI) from linear regression analysis adjusted for age.

### Physical function assessments

#### Females

At the initial visit, female participants with PMR had poorer performance compared with controls in the chair stand test and SPPB, which persisted at follow-up (Table 2). In addition, female participants with PMR had slower gait speed at the follow-up visit than controls (mean difference −0.24 m/s [95% CI −0.36, −0.12], *P* < 0.001) (Table 2). When comparing changes over time, female participants with PMR experienced a significant decline in gait speed (mean change −0.12 m/s [95% CI −0.20, −0.04], *P* = 0.007), which was significantly greater than the controls (mean difference −0.13 m/s [95% CI −0.23, −0.03], *P* = 0.009). There were no significant within-group changes or between-group differences for grip strength, upper or lower limb 1-RM strength or power amongst females, with the exception that lower limb 40% 1-RM muscle power increased significantly in participants with PMR (Table 2).

#### Males

At the initial visit, male participants with PMR compared with the controls had significantly weaker upper limb 1-RM strength and lower limb 1-RM strength, but these differences were not apparent at follow-up (Table 3). Similar results were found for 40% and 70% 1-RM muscle power (Table 3). When comparing changes over time, male participants with PMR experienced an improvement in upper limb 1-RM strength (mean change 6.3 kg [95% CI: 2.3, 10.3], *P* = 0.007), which differed significantly from the change experienced by the controls (mean difference in change scores 8.8 kg [95% CI: 3.8, 13.7], *P* = 0.001). Similarly, participants with PMR experienced a significant net benefit for the changes in 40% and 70% 1-RM muscle power compared with the controls (Table 3). There were no significant within-group changes or between-group differences for gait speed, chair stand test, SPPB or grip strength amongst males (Table 3).

### Body composition

Amongst females, there were no differences in body composition between groups at either visit (Table 4). Amongst males, participants with PMR had lower SMI than the controls at both the initial and follow-up visit (Table 5). At the follow-up visit, male participants with PMR also had lower total body lean mass than the controls, largely due to a mean 1.6 kg increase in the control group over time (Table 5). There were no significant within-group changes or between-group differences for the change over time for either sex (Tables 4 and 5).

**Table 4.** Body composition in participants with PMR vs controls (females only)^a^^,^^b^

	Initial visit^c^	Follow-up visit^c^	Within-group change^d^	Net difference for change^e^
Weight (kg)				
Control	69.86 ± 2.77	71.11 ± 3.09	1.25 (−1.41, 3.91)	−0.82 (−3.67, 2.02)
PMR	71.97 ± 3.27	72.22 ± 3.81	0.44 (−1.24, 2.12)	
Adjusted difference^f^	2.10 (−6.76, 10.96)	0.88 (−9.14, 10.91)		
BMI (kg/m^2^)				
Control	27.26 ± 0.98	27.65 ± 1.05	0.39 (−0.51, 1.28)	−0.28 (−1.26, 0.70)
PMR	28.64 ± 1.30	28.75 ± 1.36	0.11 (−0.53, 0.74)	
Adjusted difference^f^	1.39 (−1.96, 4.74)	1.09 (−2.46, 4.64)		
Total body lean mass (kg)				
Control	38.25 ± 1.24	37.55 ± 1.22	−0.70 (−1.48, 0.07)	−0.12 (−1.26, 1.03)
PMR	39.55 ± 1.43	38.74 ± 1.23	−0.82 (−1.84, 0.20)	
Adjusted difference^f^	1.26 (−2.54, 5.07)	1.15 (−2.35, 4.66)		
SMI (kg/m^2^)				
Control	6.07 ± 0.18	6.02 ± 0.18	−0.05 (−0.19, 0.09)	−0.02 (−0.23, 0.19)
PMR	6.31 ± 0.20	6.24 ± 0.18	−0.07 (−0.27, 0.12)	
Adjusted difference^f^	0.23 (−0.30, 0.77)	0.22 (−0.31, 0.74)		
Total body fat mass (kg)				
Control	30.39 ± 1.75	31.39 ± 2.09	1.00 (−0.64, 2.64)	0.24 (−2.19, 2.67)
PMR	31.58 ± 2.21	32.82 ± 2.62	1.24 (−0.93, 3.41)	
Adjusted difference^f^	1.21 (−4.59, 7.02)	1.43 (−5.50, 8.35)		
Total body fat percentage (%)				
Control	42.63 ± 1.06	43.68 ± 1.25	1.06 (−0.33, 2.44)	0.02 (−2.11, 2.16)
PMR	42.37 ± 1.79	43.45 ± 2.02	1.08 (−0.87, 3.03)	
Adjusted difference^f^	−0.20 (−4.33, 3.93)	−0.19 (−5.04, 4.66)		

a Statistical significance not achieved for any result.

b SMI: skeletal mass index = appendicular lean mass/height^2^.

c Inferred mean ± standard error in each group from multiply imputed dataset.

d Mean change scores (95% CI) in each group from a linear regression analysis.

e Net difference in change scores (95% CI) from a linear mixed-effects model.

f Mean difference (95% CI) from linear regression analysis adjusted for age.

**Table 5.** Body composition in participants with PMR vs controls (males only)^a^^,^^b^

	Initial visit^c^	Follow-up visit^c^	Within-group change^d^	Net difference for change^e^
Weight (kg)				
Control	88.18 ± 4.28	86.49 ± 3.92	−1.69 (−4.33, 0.96)	0.48 (−2.08, 3.04)
PMR	82.74 ± 3.90	83.25 ± 3.91	−1.25 (−2.79, 0.28)	
Adjusted difference^e^	−8.69 (−19.75, 2.37)	−5.97 (−17.24, 5.29)		
BMI (kg/m^2^)				
Control	29.31 ± 1.01	28.78 ± 1.00	−0.53 (−1.35, 0.30)	0.25 (−0.66, 1.15)
PMR	27.64 ± 1.29	27.36 ± 1.28	−0.28 (−0.94, 0.38)	
Adjusted difference^e^	−2.42 (−5.86, 1.02)	−2.00 (−5.49, 1.49)		
Total body lean mass (kg)				
Control	55.42 ± 3.13	57.02 ± 2.36	1.60 (−2.94, 6.14)	−2.20 (−5.81, 1.42)
PMR	53.32 ± 1.87	52.72 ± 2.11	−0.59 (−1.69, 0.50)	
Adjusted difference^e^	−3.69 (−10.59, 3.22)	−6.34 (−12.11, −0.58)^*^		
SMI (kg/m^2^)				
Control	8.23 ± 0.26	8.24 ± 0.28	0.01 (−0.16, 0.18)	0.01 (−0.20, 0.23)
PMR	7.43 ± 0.23	7.45 ± 0.27	0.02 (−0.15, 0.19)	
Adjusted difference^e^	**−1.08 (−1.65, −0.51)^‡^**	**−1.09 (−1.75, −0.43)** ^†^		
Total body fat mass (kg)				
Control	28.09 ± 1.90	27.22 ± 1.91	−0.86 (−2.70, 0.97)	0.06 (−2.16, 2.29)
PMR	27.88 ± 2.25	27.09 ± 2.25	−0.80 (−2.51, 0.91)	
Adjusted difference^e^	−1.55 (−7.64, 4.55)	−1.10 (−7.44, 5.23)		
Total body fat percentage (%)				
Control	31.46 ± 0.93	30.96 ± 1.09	−0.50 (−1.63, 0.63)	0.24 (−1.67, 2.16)
PMR	32.45 ± 1.37	32.19 ± 1.34	−0.26 (−1.98, 1.46)	
Adjusted difference^e^	0.68 (−3.04, 4.40)	1.19 (−2.66, 5.04)		

a Statistically significant results are highlighted in bold (*P ≤ 0.05, ^†^*P* ≤ 0.01, ^‡^*P* ≤ 0.001).

b SMI: Skeletal mass index = appendicular lean mass/height^2^.

c Inferred mean ± standard error in each group from multiply imputed dataset.

d Mean change scores (95% CI) in each group from a linear regression analysis.

e Net difference in change scores (95% CI) from a linear mixed-effects model.

f Mean difference (95% CI) from linear regression analysis adjusted for age.

### Association with cumulative glucocorticoid exposure

Amongst male participants with PMR, change in weight from initial to follow-up visit was associated with cumulative glucocorticoid exposure, although this did not reach statistical significance (*ß* coefficient 1.28 [95% CI: −0.01, 2.57], *P* = 0.052). There were no other significant associations between changes in physical function or body composition and cumulative glucocorticoid exposure for either males or females (Supplementary Tables S2 and S3).

### Frailty

**Figure 1.**
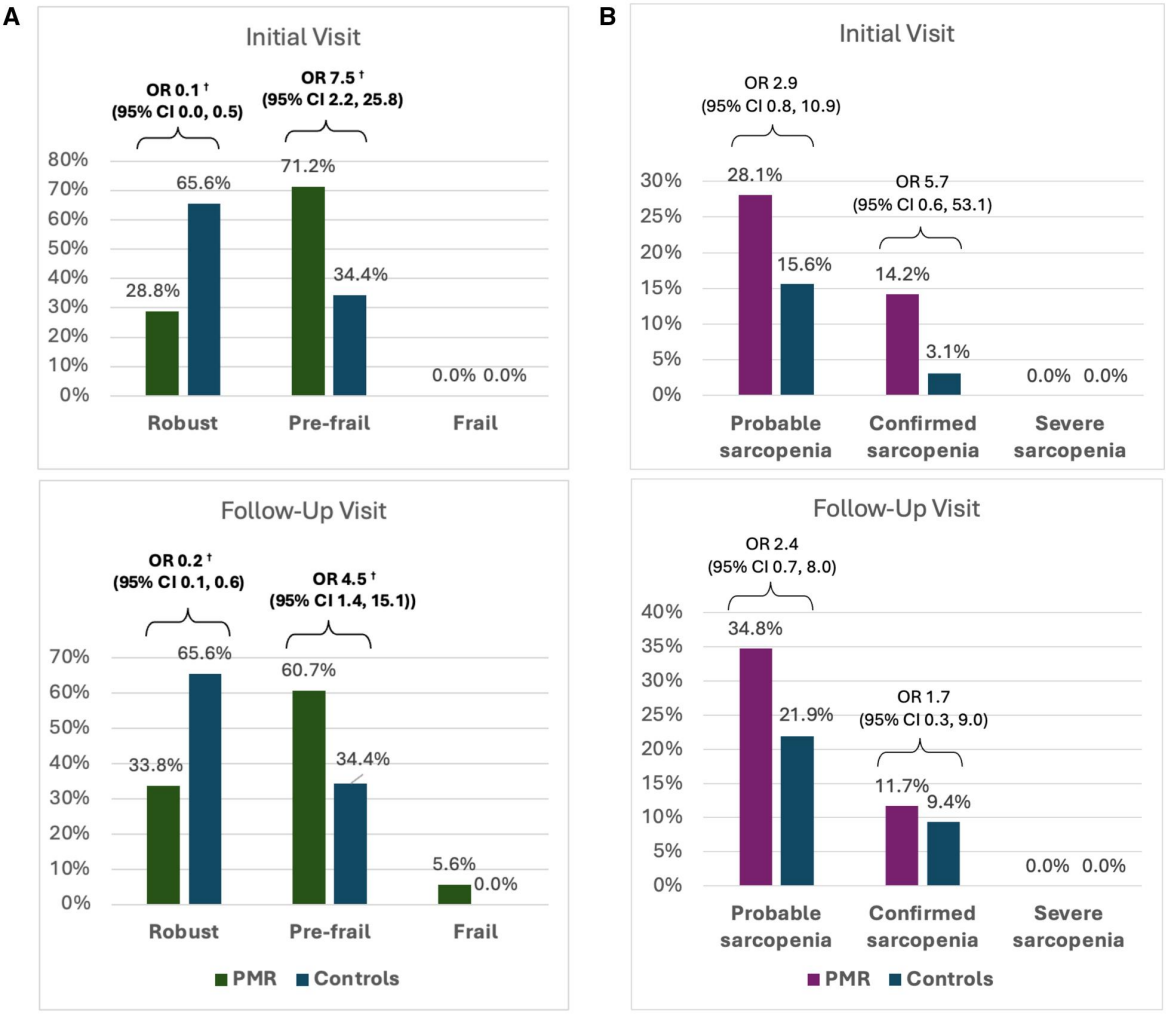
Rates of (**A**) frailty and (**B**) sarcopenia in participants with PMR vs controls at the initial and follow-up visit. Frailty defined as per Fried’s frailty phenotype. Sarcopenia defined as per EWGSOP2 criteria. Values are presented as proportion and odds ratio (95% CI). Proportions shown without absolute values as figures derived from multiply imputed dataset. Odds ratios derived from logistic regression analysis adjusted for age and sex. Statistically significant results are highlighted in bold (*P ≤ 0.05, ^†^*P* ≤ 0.01, ^‡^*P* ≤ 0.001)

At the initial visit, 71.2% [95% CI: 55.9, 86.5] of participants with PMR were considered pre-frail compared with 34.4% [95% CI: 17.6, 51.1] of the controls, resulting in an odds ratio of 7.5 [95% CI: 2.2, 25.8], *P* = 0.001. No participants were frail in either group at the initial visit (Fig. 1). At the follow-up visit, 60.7% [95% CI: 43.8, 77.5] of participants with PMR were considered pre-frail compared with 34.4% [95% CI: 17.6, 51.1] of the controls, resulting in an odds ratio of 4.5 [95% CI: 1.4, 15.1], *P* = 0.01. The proportion of participants with PMR considered frail at follow-up was 5.6%; no controls were considered frail (Fig. 1). The most commonly fulfilled criterion for frailty in the PMR group was exhaustion (present in 50.0% and 58.3% at the initial and follow-up visits, respectively), followed by weakness (38.4% and 30.7%) (Supplementary Table S4).

### Sarcopenia

At the initial visit, 28.1% [95% CI: 13.0, 43.1] of the PMR cases had probable sarcopenia compared with 15.6% [95% CI: 2.8, 28.4] of the controls. Confirmed sarcopenia was present in 14.2% [95% CI: 2.4, 25.9] of the PMR cases and 3.1% [95% CI: −3.0, 9.3] of the controls. However, the difference between the cases and the controls was not statistically significant (Fig. 1). At the follow-up visit, 34.8% [95% CI: 18.2, 51.4] of the PMR cases had probable sarcopenia compared with 21.9% [95% CI: 7.3, 36.5] of the controls, with confirmed sarcopenia present in 11.7% [95% CI: 0.7, 22.6] of the PMR cases and 9.4% [95% CI: −0.9, 19.7] of the controls; there were no significant between-group differences (Fig. 1). No participants in either group had severe sarcopenia at either study visit.

## Discussion

In the first cohort study to examine changes to muscle function and body composition in patients with PMR compared with the controls, we report a novel finding of disproportionately declining gait speed in females with PMR compared with the controls. Both male and female patients with PMR also had lower functional independence (HAQ-DI) than the controls at both timepoints despite low disease activity. Female participants with PMR additionally had poorer physical performance for measures related to gait speed, functional strength and balance, and male participants with PMR had lower muscle strength and power at the initial visit compared with controls. Taken together, these results demonstrate the lasting impact of PMR on physical performance despite apparent disease control using conventional disease measures, particularly amongst females.

Previous studies of functional independence in PMR cohorts have reported mean HAQ-DI scores that improve following treatment but do not entirely normalize [19, 20]. This has been attributed in part to the presence of comorbidities [19]. However, our study had the advantage of including a control group that was well matched in terms of comorbidity burden and habitual physical activity level, and yet participants with PMR were still found to have greater disability. From our study design it is not possible to ascertain the level of functional independence of PMR cases prior to their diagnosis of PMR, but our findings raise the possibility of persistent disability arising as a consequence of the disease itself. Aside from being an aspect of the condition that is a priority to patients, dependence of ADLs is associated with both morbidity and mortality, thus warranting close attention from treating clinicians [21].

Gait speed is an important measure of physical performance in older adults that predicts disability with ADLs, cognitive impairment, falls, institutionalization and mortality [22, 23]. A decline in gait speed of ≥0.05 m/s is considered a clinically important change [24]. In our study, the mean decline in gait speed in female participants with PMR was 0.12 m/s over the 18-month follow-up period, which would be classified as a clinically meaningful change that may increase the risk for falls and other adverse health-related outcomes. Furthermore, female participants with PMR in our study performed more poorly on measures of lower limb functional strength, mobility and balance (chair stand test and SPPB) than the controls at the 18-month follow-up. Like gait speed, these measures have important functional correlates including worsening ADL dependence, falls, frailty and mortality [25–27]. Such findings highlight the detrimental effects of PMR and its treatment, contradicting the long-held perception of the condition being a benign and self-limiting entity.

The reason(s) for the heterogeneous physical function findings between males and females is uncertain. One possible explanation is that muscle tissue is sexually dimorphic, with greater muscle atrophy occurring in females than males after glucocorticoid exposure [28, 29]. In part support of this finding, Sattui *et al.* found that female sex was a risk factor for frailty in PMR [10]. Another potential hypothesis relates to inflammation, specifically interleukin (IL)-6 levels. Higher levels of IL-6 promote catabolic processes and are associated with declining gait speed [30, 31]. Production of IL-6 is partly regulated by oestrogen and testosterone [31]. Whilst females experience a rapid reduction in oestrogen production during menopause, up to 80% older men still retain normal levels of testosterone [32]. Furthermore, a meta-analysis demonstrated a correlation between high IL-6 and weak grip strength amongst both sexes, but males had significantly higher IL-6 levels than females despite better muscle strength, suggesting sex differences in the absolute effect of IL-6 on muscle [33]. As a key cytokine in the pathogenesis of PMR, it is possible that the inflammatory milieu of PMR contributes to IL-6-driven physical decline and potentiates the differences between male and female muscle outcomes [34].

Another unique aspect of our study is that we examined body composition, but we did not find any significant differences in change over time between the groups, although there was some evidence for lower lean mass in male participants with PMR compared with the controls. Furthermore, we observed numerically higher rates of sarcopenia in participants with PMR than the controls, but the difference was not statistically significant, which may be explained by the low study numbers and inadequate study power rather than a true lack of difference. This is an important area for future research as sarcopenia is associated with numerous adverse health outcomes including disability, hospitalization and death [35–37], and in patients with PMR, this process may be accelerated by the catabolic effects of glucocorticoids and systemic inflammation [38, 39].

Finally, our study found a significantly higher rate of pre-frailty among participants with PMR compared with the controls (71.0% vs 34.4% and 61.7% vs 34.4% at the initial and follow-up visits, respectively). Only one previous study has examined frailty in a cross-sectional cohort of 41 patients with PMR who were in remission or had mild disease, finding a similar pre-frailty rate of 59% but a higher rate of frailty (17% vs 0–5.6% in our study) [10]. These differences may relate to modifications to the frailty criteria used in our study design, particularly to the weight loss, exhaustion, and low activity domains. In both studies, weakness and exhaustion were the two most common frailty criteria fulfilled by participants with PMR. As discussed above, inflammation is hypothesized to play a role in the pathogenesis of frailty, with IL-6 levels being 3-fold higher in frail vs non-frail older adults [40]. Inflammation may therefore contribute to the higher risk of frailty seen among patients with PMR.

Ultimately, our results highlight the detrimental effects of PMR on physical function that particularly affects females. This provides a clear rationale for targeted muscle conditioning exercises to be incorporated into the management of PMR, as this has demonstrated benefit to muscle mass, function and frailty in other rheumatic diseases [41]. Further research to evaluate the effectiveness of such a program in a PMR population is warranted.

Our study has a number of key strengths. We included a well-curated cohort of patients with PMR who all fulfilled validated classification criteria as well as having had their diagnosis confirmed clinically by a rheumatologist. We also included a well-matched control comparator group, and the prospective design of our study allowed longitudinal outcomes to be examined. To the best of our knowledge, this is the first study to investigate changes to physical function and body composition in PMR over time. However, there are several limitations that must be considered when interpreting the findings. As this was a pilot study, the sample size was determined on a pragmatic basis and was further curtailed by the COVID-19 pandemic. Participants were recruited from a tertiary referral centre, which may limit generalizability to the wider PMR population who are frequently managed in primary care. Furthermore, a modified version of Fried’s frailty criteria was used, to align with available data in our study. However, this is common practice even by the original authors themselves [42].

## Conclusion

In this prospective cohort study of patients with recently diagnosed PMR compared with age- and sex-matched individuals without PMR, we identified a disproportionate decline in gait speed over time that affected female but not male participants with PMR. At the follow-up visit 21 months after the initiation of glucocorticoid treatment, female participants with PMR also had poorer physical function (gait speed, chair stand test and SPPB) than the controls, and both male and female participants with PMR had lower functional independence (HAQ-DI). Collectively, these findings highlight the sustained detrimental impact of PMR on physical function despite appropriate treatment and apparent disease control. Future studies should look to develop a targeted muscle conditioning program that could be incorporated into routine care for patients with PMR.

## Supplementary Material

keaf375_Supplementary_Data

## Data Availability

The data underlying this article will be shared on reasonable request to the corresponding author, after Institutional Review Board approval and completion of a data user agreement.
