# Supplementary material for: Changes to physical function and body composition during the first 2 years of polymyalgia rheumatica
Source: Rheumatology (Oxford). 2025 Jul 11;64(11):5834–43. doi: 10.1093/rheumatology/keaf375 (PMC12596070; doi:10.1093/rheumatology/keaf375)
Supplement: keaf375_Supplementary_Data [file keaf375_supplementary_data.docx]

## Supplementary File

**Changes to physical function and body composition during the first two years of polymyalgia rheumatica**

Jessica L Leung^1,2^, Belinda De Ross^3^, Jenny Gianoudis^3^, Natalie Deeble^1^, Victor Yang^1^, David FL Liew^1,2^, Robin M Daly^3^, Russell RC Buchanan^1,2^, Claire E Owen^1,2^

### Contents

1. Supplementary Methods
2. Supplementary Results
3. Supplementary Tables
4. References

### Supplementary Methods (Supplementary Data S1)

**Exclusion criteria**

Exclusion criteria for cases and controls included active malignancy, current infection, neuromuscular disease, other inflammatory/autoimmune conditions requiring immunosuppression, or chronic pain syndromes. Patients with PMR were additionally excluded if they had received treatment with disease-modifying antirheumatic drugs (DMARDs) prior to or at the initial visit, and controls were excluded if they had any condition requiring regular administration of systemic glucocorticoids.

**Grip strength**

Grip strength (kg) was measured with the elbow at 90 degrees of flexion, using a hand-held device (Jamar dynamometer, Asimov Engineering Co., Los Angeles, CA, USA). After one practice trial, the higher value of two trials from the dominant hand was used for analysis.

**Maximum lower limb strength and power**

Lower limb muscle strength was measured using a bilateral leg press on Keiser A420 pneumatic equipment (Fresno, CA, USA). Before the assessment, participants completed a 2-minute warm-up using a stationary exercise bike. To determine 1-RM, each participant first performed a practice set of eight repetitions at a load of 50% body weight. After a 1-minute rest, participants then completed a further five repetitions at a load of 100% body weight. The weight was then increased incrementally until only one repetition with correct technique could be completed, with a 2-minute rest before each attempt. The heaviest load achieved was recorded as the one-repetition maximum weight (1-RM, in kg).

After a 15-minute rest, leg press peak muscle power was assessed. First, participants undertook a warm-up set of five repetitions at 50% of body weight. Participants were then instructed to complete five repetitions with the concentric portion of each repetition performed as fast as possible. The maximum power output achieved at 40% and 70% 1-RM was recorded as peak power.

**Maximum upper limb strength**

Upper limb muscle strength was measured using a seated row on Keiser A420 pneumatic equipment (Fresno, CA, USA). Before the assessment, participants completed a warm-up of five repetitions at a load of 15% body weight. After a 1-minute rest, the weight was increased incrementally until only one repetition with correct technique could be completed, with a 2-minute rest between each attempt. The heaviest load achieved was recorded as the 1-RM (kg).

**Habitual physical activity and sedentary time**

Self-reported daily physical activity was assessed using the Physical Activity Scale for the Elderly (PASE) survey, which is a validated questionnaire that records time spent in sedentary and physical activities during the prior seven days [16]. Objectively measured physical activity (average daily step count) and sedentary time (minutes) was assessed using an accelerometer (ActivPAL3, PAL technologies Ltd, Glasgow, UK) attached to the anterior right thigh for a seven-day period. A minimum of 12 waking hours of wear time, for a minimum of five days, was required for data to be included.

**Sarcopenia**

A participant had probable sarcopenia if low muscle strength was demonstrated (either grip strength <27kg for males or <16kg for females, and/or five-times sit-to-stand time >15 seconds). Sarcopenia was considered confirmed if a participant had low muscle strength in addition to low SMI (< 7.0 kg/m^2^ in males or < 5.5 kg/m^2^ in females). Severe sarcopenia was considered if a participant also had low gait speed (≤ 0.8 m/sec) and/or a low SPPB score (≤ 8 points).

**Missing Data**

Multiple imputation (MI) was used to handle missing data. Gender, age, and BMI at baseline were included as predictors in the imputation model for all variables, along with the value of the variable being imputed at any other time point where it was measured. MI was conducted using the Stata command *mi impute chained*, with 30 imputed datasets created using a burn-in of 10 iterations, stratified by participant group.

### Supplementary Results (Supplementary Data S2)

**Habitual physical activity and sedentary time**

There was no significant difference in the average daily step count between participants with PMR and controls at the initial visit (mean difference 162 [95% CI -1510, 1834] p=0.85) or follow-up visit (mean difference -755 [95% CI -2547, 1036] p=0.40). There was also no significant difference in the average daily sedentary time between groups (mean difference -36.3 [95% CI -92.3, 19.6] p=0.20 and -20.4 [95% CI -73.6, 32.7] p=0.45 at the initial and follow-up visits respectively).

### Supplementary Tables

| **Supplementary Table S1 – Modified criteria used for the diagnosis of frailty (based on Fried’s frailty phenotype)** |
| --- |

| **Frailty characteristic** | **Criteria** |
| --- | --- |
| **Weight loss ^a^** | BMI ≤ 18.5 |
| **Weakness** | Males:   - BMI ≤ 24 and grip strength < 29kg - BMI 24.1 – 28 and grip strength < 30kg - BMI > 28 and grip strength < 32kg   Females:   - BMI ≤ 23 and grip strength < 17kg - BMI 23.1 – 26 and grip strength < 17.3kg - BMI 26.1 – 29 and grip strength < 18kg - BMI > 29 and grip strength < 21kg |
| **Exhaustion ^a^** | SF-36 Vitality Scale questions “How much of the time during the past 4 weeks did you feel (i) worn out, and (ii) tired?”  The following responses fulfilled the criterion   - “a lot of the time” - “most of the time” - “all of the time” |
| **Slowness** | Males:   - Height ≤ 173 cm and gait speed ≤ 0.6531 m/s - Height > 173 cm and gait speed ≤ 0.7620 m/s   Females:   - Height ≤ 159 cm and gait speed ≤ 0.6531 m/s - Height > 159 cm and gait speed ≤ 0.7620 m/s |
| **Low activity ^a^** | Males:   - PASE score ≤ 30.0 points ^b^   Females:   - PASE score ≤ 27.5 points ^b^ |
| a) Modifications made to criteria to align with available data  b) PASE thresholds used have demonstrated similar criteria fulfilment rates to Fried’s original definition [1] | |

| **Supplementary Table S2 - Association between cumulative glucocorticoid exposure and change in physical function or body composition parameter from initial to follow-up visit (females only)** |
| --- |

|  | **ß Coefficient ^a^** | **95% CI** | **p-value** |
| --- | --- | --- | --- |
| **Physical function parameters ^b^** | | | |
| **HAQ-DI** | 0.09 | -0.23, 0.41 | 0.55 |
| **Gait speed (m/sec)** | -0.01 | -0.12, 0.10 | 0.81 |
| **Chair stand test (sec)** | 1.21 | -1.95, 4.38 | 0.41 |
| **SPPB** | -0.9 | -2.1, 0.3 | 0.12 |
| **Grip strength (kg)** | -2.2 | -5.9, 1.5 | 0.22 |
| **Upper limb 1-RM strength (kg)** | -1.1 | -4.9, 2.8 | 0.55 |
| **Lower limb 1-RM strength (kg)** | 4.6 | -25.7, 34.9 | 0.73 |
| **Lower limb peak muscle power at 40% 1-RM (W)** | -2.3 | -97.1, 92.5 | 0.96 |
| **Lower limb peak muscle power at 70% 1-RM (W)** | 1.2 | -125.8, 128.2 | 0.98 |
| **Body composition parameters ^c^** | | | |
| **Weight (kg)** | 0.29 | -1.45, 2.04 | 0.72 |
| **BMI (kg/m^2^)** | 0.10 | -0.56, 0.76 | 0.75 |
| **Total body lean mass (kg)** | -358.98 | -1525.54, 807.59 | 0.51 |
| **SMI (kg/m^2^)** | 0.03 | -0.22, 0.27 | 0.81 |
| **Total body fat mass (kg)** | 816.27 | -1818.00, 3450.53 | 0.50 |
| **Total body fat percentage (%)** | 1.13 | -1.23, 3.49 | 0.31 |
| a) Coefficients derived from linear regression analysis adjusted for age  b) HAQ-DI: Health Assessment Questionnaire-Disability Index; SPPB: Short Physical Performance Battery; 1-RM: 1-repetition maximum weight  c) BMI: Body mass index; SMI: Skeletal mass index = appendicular lean mass / height^2^ | | | |

| **Supplementary Table S3 - Association between cumulative glucocorticoid exposure and change in physical function or body composition parameter from initial to follow-up visit (males only)** |
| --- |

|  | **ß Coefficient ^a^** | **95% CI** | **p-value** |
| --- | --- | --- | --- |
| **Physical function parameters ^b^** | | | |
| **HAQ-DI** | 0.06 | -0.09, 0.20 | 0.41 |
| **Gait speed (m/sec)** | -0.07 | -0.15, 0.02 | 0.12 |
| **Chair stand test (sec)** | 0.16 | -1.54, 1.87 | 0.83 |
| **SPPB** | 0.0 | -0.5, 0.6 | 0.94 |
| **Grip strength (kg)** | -0.5 | -2.5, 1.6 | 0.64 |
| **Upper limb 1-RM strength (kg)** | -0.8 | -4.7, 3.1 | 0.62 |
| **Lower limb 1-RM strength (kg)** | -12.7 | -43.0, 17.7 | 0.37 |
| **Lower limb peak muscle power at 40% 1-RM (W)** | 28.8 | -56.3, 114.0 | 0.45 |
| **Lower limb peak muscle power at 70% 1-RM (W)** | 33.3 | -112.3, 178.9 | 0.59 |
| **Body composition parameters ^c^** | | | |
| **Weight (kg)** | 1.28 | -0.01, 2.57 | 0.052 |
| **BMI (kg/m^2^)** | 0.17 | -0.49, 0.83 | 0.58 |
| **Total body lean mass (kg)** | 131.52 | -951.32, 1214.37 | 0.79 |
| **SMI (kg/m^2^)** | 0.05 | -0.10, 0.21 | 0.45 |
| **Total body fat mass (kg)** | 1220.78 | -337.08, 2778.63 | 0.11 |
| **Total body fat percentage (%)** | 0.67 | -1.08, 2.42 | 0.41 |
| a) Coefficients derived from linear regression analysis adjusted for age  b) HAQ-DI: Health Assessment Questionnaire-Disability Index; SPPB: Short Physical Performance Battery; 1-RM: 1-repetition maximum weight  c) BMI: Body mass index; SMI: Skeletal mass index = appendicular lean mass / height^2^ | | | |

| **Supplementary Table S4 – Frequency of frailty criteria fulfilled by participants with PMR and controls** |
| --- |

| **Frailty Criterion** | **PMR** | | **Controls** | |
| --- | --- | --- | --- | --- |
|  | **Baseline** | **Follow-Up** | **Baseline** | **Follow-Up** |
| **Weight loss (%)** | 5.6  (-2.1, 13.2) | 3.4  (-3.1, 10.0) | 0  (0, 0) | 0  (0, 0) |
| **Weakness (%)** | 38.4  (21.8, 55.1) | 30.7  (15.0, 46.3) | 28.1  (12.3, 44.0) | 31.3  (14.9, 47.6) |
| **Exhaustion (%)** | 50.0  (33.4, 66.6) | 58.3  (41.9, 74.7) | 15.6  (2.8, 28.4) | 9.4  (0, 19.7) |
| **Slowness (%)** | 2.8  (-2.7, 8.3) | 2.8  (-2.7, 8.3) | 0  (0, 0) | 0  (0, 0) |
| **Low activity (%)** | 2.8  (-2.7, 8.3) | 2.8  (-2.7, 8.3) | 0  (0, 0) | 0  (0, 0) |
| Values are presented as proportion (95% CI) without absolute values, as figures are derived from a multiply imputed dataset. | | | | |

### References

1. Ziller C, Braun T, and Thiel C, *Frailty Phenotype Prevalence in Community-Dwelling Older Adults According to Physical Activity Assessment Method.* Clin Interv Aging, 2020. **15**: p. 343-355.
